# Efficacy of doxycycline therapy for macrolide-resistant *Mycoplasma pneumoniae* pneumonia in children at different periods

**DOI:** 10.1186/s13052-024-01615-y

**Published:** 2024-03-05

**Authors:** Ying Chen, Yan Zhang, Qiong-Ni Tang, Hong-Bo Shi

**Affiliations:** Pediatric Department, Ningbo Medical Center Lihuili Hospital, Ningbo, Zhejiang China

**Keywords:** Macrolide-resistant, Mycoplasma pneumoniae, Doxycycline

## Abstract

**Background:**

The prevalence of macrolide-resistant Mycoplasma pneumoniae has increased considerably. Treatment in children has become challenging. This study aimed to evaluate the efficacy of doxycycline therapy for macrolide-resistant Mycoplasma pneumoniae pneumonia in children at different periods.

**Methods:**

We retrospectively analyzed the data of patients with macrolide-resistant *Mycoplasma pneumoniae* pneumonia hospitalized between May 2019 to August 2022. According to treatment, patients were divided into three groups: oral doxycycline treatment alone (DOX group), changed from intravenous azithromycin to oral doxycycline (ATD group), and intravenous azithromycin treatment alone (AZI group). ATD group cases were separated into two sub-groups: intravenous azithromycin treatment<3 days (ATD1 group) and ≥ 3 days (ATD2 group). Clinical symptoms were compared in each group and adjusted by Propensity score matching (PSM) analysis.

**Results:**

A total of 106 were recruited in this study. 17 (16%) were in DOX group, 58 (55%) in ATD group, and 31(29%) in AZI group. Compared with ATD group and AZI group, the DOX group showed shorter hospitalization duration and fever duration after treatment, while higher rate of chest radiographic improvement. After using PSM analysis, shorter days to hospitalization duration (*P* = 0.037) and to fever duration after treatment (*P* = 0.027) in DOX + ATD1 group than in ATD2 group was observed. A higher number of patients in the DOX + ATD1 group achieved defervescence within 72 h (*P* = 0.031), and fewer children received glucocorticoid adjuvant therapy (*P* = 0.002). No adverse reactions associated with doxycycline was observed during treatment.

**Conclusions:**

Children receiving early oral doxycycline had a shorter duration of fever and hospitalization in macrolide-resistant *Mycoplasma pneumoniae* patients.

## Background

*Mycoplasma pneumoniae* (*M. pneumoniae*) is one of the most common causes of upper and lower respiratory tract infections, particularly in children and young adults. The majority of *M. pneumoniae* pneumonia are benign and self-limiting disease. However, some patients may develop severe *M. pneumoniae* pneumonia or refractory *M. pneumoniae* pneumonia, causing progressive pneumonia or various extrapulmonary complications [1]. These cases may be related to the occurrence of macrolide-resistant (MR) *M. pneumoniae* [2, 3]. This resistance is associated with point mutations in the V region of the 23S rRNA gene and leads to high-level resistance to macrolides [4]. Therefore, the efficacy of macrolide treatment was shown to be lower in patients infected with macrolide-resistant isolates than in patients infected with macrolide-sensitive isolates [5, 6].

In recent years, the global patterns in the proportion of MR *M. pneumoniae* infections showed an increasing trend, and the proportion of MR *M. pneumoniae* infections was highest in China [3, 7]. Treatment with the increase in MR *M. pneumoniae* has become challenging. Because this resistance may lead to more extrapulmonary complications and severe clinical features [2], alternative antibiotic treatment can be required, including tetracyclines or fluoroquinolones. To date, no tetracycline resistance has been reported in *M. pneumoniae* clinical isolates. In vitro antimicrobial susceptibility testing showed that *M. pneumoniae* in all cases was sensitive to tetracyclines, including doxycycline and minocycline [8].

MR *M. pneumoniae* pneumonia is characterized by an excessive immune response against the pathogen as well as direct injury caused by an increasing *M. pneumoniae* load [9]. Study indicates that children with higher *M. pneumoniae* abundance in the bronchoalveolar lavage fluid tend to have a longer hospital stay and higher fever peak [10]. This suggests that the loading of *M. pneumoniae* is associated with clinical severity [11]. Doxycycline, as an alternative drug for treating MR *M. pneumoniae*, can inhibit the replication of *M. pneumoniae* DNA and reduce the load of pulmonary pathogens [12]. However, the exact timing of doxycycline treatment has not been established at present. In this study, we aimed to evaluate the efficacy of doxycycline therapy for macrolide-resistant Mycoplasma pneumoniae pneumonia in children at different periods.

## Materials and methods

### Study subjects

We retrospectively reviewed the medical records of children without prior underlying diseases with Community acquired pneumonia hospitalized at the Ningbo Medical Center Lihuili Hospital between May 2019 and August 2022. All the evaluated patients had signs and symptoms indicative of pneumonia, such as fever, cough, and abnormal chest radiographic findings compatible with pneumonia [13]. The *M. pneumoniae* infection was determined by polymerase chain reaction test of nasopharyngeal aspirates obtained from the patients on admission. Samples positive for *M. pneumoniae* were subjected to direct DNA sequencing of the domain V of the 23S rRNA gene to identify A2063G or A2064G mutation site using real-time fluorescent PCR assay kit (Jiangsu Mole Bioscience Co., Ltd, China) in accordance with the manufacturer’s instructions. It took about 2 days to determine whether there is a resistance mutation.

Exclusion criteria were as follow: (1) other pathogens were found before treatment, including bacteria, respiratory syncytial virus, influenza viruses, parainfluenza viruses, coronaviruses, human rhinoviruses, adenoviruses, human metapneumovirus and chlamydia pneumoniae; (2) patients who had been started on treatment for macrolide or doxycycline prior to admission; (3) children who have chronic disease (such as tuberculosis, asthma and immunodeficiency) states predisposing them to recurrent lung infections; (4) discharge within 48 h after enrollment and insufficient data; (5) children younger than 8 years were excluded because they could not be treated with doxycycline.

### Methods

In our study, all patients were treated with intravenous azithromycin or oral doxycycline. The dosage of intravenous azithromycin was 10 mg/kg once daily, and oral doxycycline was administered once every 12 h at doses of 2.2 mg/kg, in accordance with the package insert accompanying each drug [13]. The primary antibiotic selection was made by the attending pediatrician. Oral doxycycline should be used if the patient had a history of exposure to MR *M. pneumoniae*. The preferred treatment for *M. pneumoniae* pneumonia is macrocyclic antibiotics [13]. Therefore, before the results of pathogen and *M. pneumoniae* mutation site testing are available, intravenous azithromycin was chosen as the primary antibiotic for patients with suspected *M. pneumoniae* pneumonia. After obtaining evidence of MR *M. pneumoniae* infection, some patients were changed from intravenous azithromycin to oral doxycycline, while others continued treatment with intravenous azithromycin due to inability to tolerate swallowing capsules. According to treatment, these patients were divided into three groups: oral doxycycline treatment alone (DOX group), changed from intravenous azithromycin to oral doxycycline (ATD group) and intravenous azithromycin treatment alone (AZI group). ATD group cases were separated into two sub-groups: intravenous azithromycin treatment<3 days (ATD1 group) and intravenous azithromycin treatment ≥ 3 days (ATD2 group).

### Data collection

Clinical information was retrospectively collected from the medical records of the patients. The collected data included demographics, hospitalization period, duration of fever (febrile days before macrolide or doxycycline treatment, febrile days after treatment, time to defervescence), laboratory results upon admission, chest radiographic findings and adverse reactions during treatment. The duration of fever was defined as the number of days for which the patient had a body temperature of ≥ 38℃ with an interval of <24 h between each episode of fever. Defervescence of fever was defined as a decline in body temperature up to < 37.5℃ for > 48 h. All patients underwent chest radiographic examination before admission, and a second chest radiographic examination was performed 7 to 10 days after treatment. The chest radiographic findings were from the records read by two radiologists and classified according to the presence of consolidation lobar, patchy and effusion. If the results of the patient’s X-rays showed that reduction of more than 30% in consolidation and infiltration area compared to before treatment, we consider the patient to be a consolidation and/or infiltration absorption case.

### Statistical analysis

SPSS 25.0 statistical software was applied for Propensity score matching (PSM) and analysis. The data were expressed as median (IQR) for continuous variables or as number of cases (percentage) of a specific group for categorical variables. The Kruskal-Wallis test was used for continuous variables. If the variables were statistically significant when compared among more than two groups, they were further analyzed by Mann-Whitney U test for comparing two groups. The Pearson’s Chi-squared or Fisher’s exact test were used for categorical variables. To reduce the effect of possible selective bias, patients in the DOX + ATD1 and ATD2 groups, were matched with those in non-biopsy group for a 1:1 PSM with a caliper value of 0.02. Matching factors included age, gender, fever duration prior to treatment, and chest radiographic before admission. Two-sided p-value < 0.05 was considered to be statistically significant.

## Results

### Demographic and clinical characteristics

**Fig. 1:**
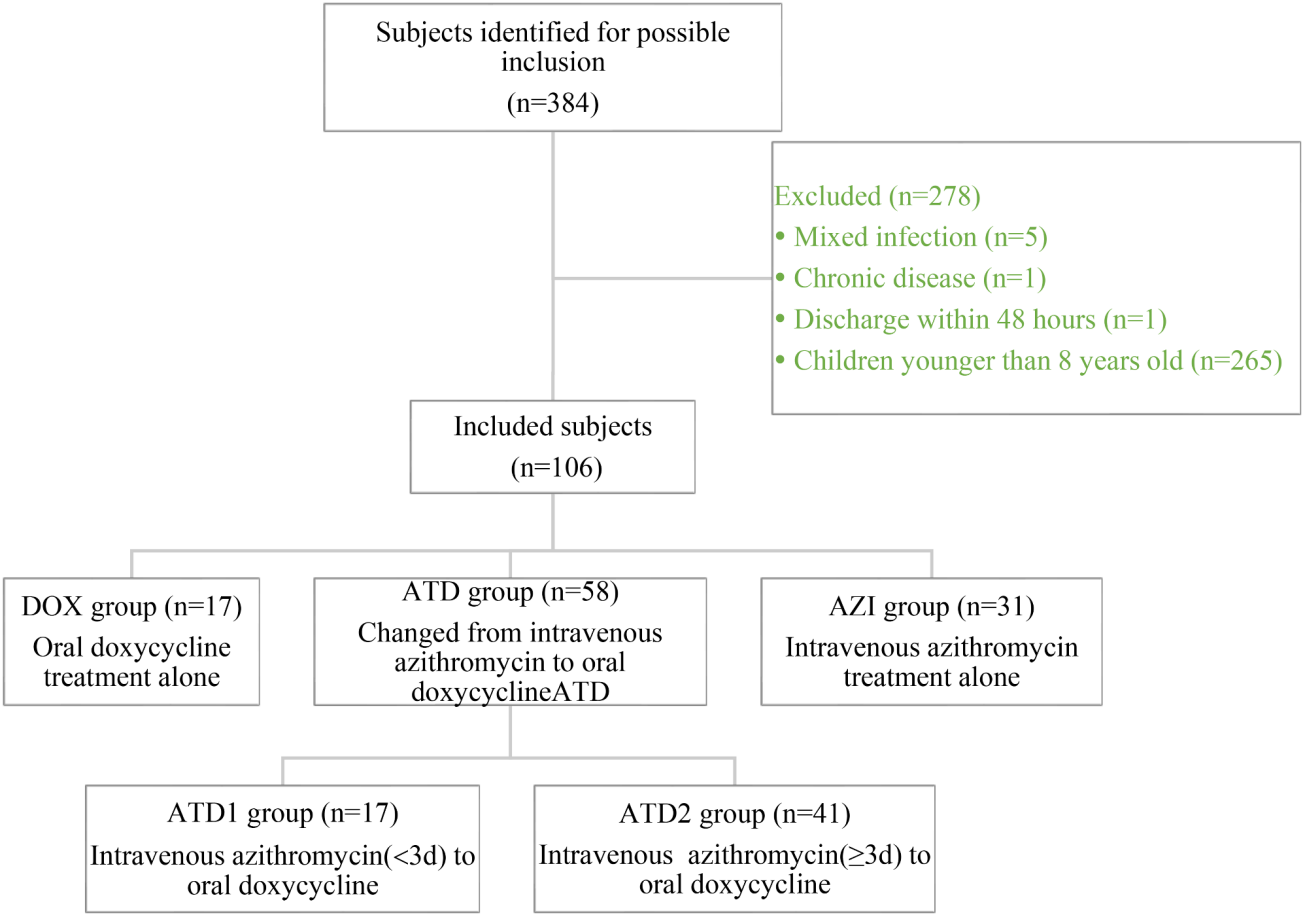
Flow chart for the inclusion and classification of the study subjects

The total number of hospitalized patients was 5589, who were tested for *M. pneumoniae* PCR tests of the nasopharyngeal aspirates between May 2019 to August 2022 due to clinically suspected *M. pneumoniae* infection. Among the cases tested, 10% (533/5589) were *M. pneumoniae* PCR positive, and 72% (384/533) showed point mutations in domain V of 23S rRNA. The prevalence rate of patients tests positive for MR *M. pneumoniae* infection peaked in summer and autumn season of 2019 during the study period. Of the patients with MR *M. pneumoniae* pneumonia, we excluded 278 patients following the exclusion criteria. Consequently, a total of 106 were recruited in this study. 17 (16%) were in DOX group, 58 (55%) in ATD group, and 31(29%) in AZI group (Fig. 1).

The demographics and clinical characteristics were shown in Table 1. The median age of patients in three groups was 10 (8.5, 11) years old, 9 (8.75, 10) years old, and 9 (9, 9) years old, respectively. The number of males in DOX, ATD, and AZI groups was 5 (29%), 32 (55%), and 18 (58%), and the number of febrile patients was 15 (88%), 55 (95%), and 31 (100%), respectively. Fever duration prior to treatment showed no differences compared among the three groups (*P* = 0.114). No statistically significant differences were found among the groups for the occurrence of pleural effusion (DOX 35%, ATD 26%, AZI 42%) (*P* = 0.288). There were no statistically significant differences among the three groups in terms of laboratory data, including white blood cell count, neutrophil percentage, platelets, C-reactive protein, procalcitonin and lactate dehydrogenase (*P* > 0.05).

**Table 1:** Characteristics of patients with macrolide-resistant MP pneumonia in each treatment group

	DOX group	ATD group	AZI group	p-value*
	n = 17	n = 58	n = 31	
Age, years	10 (8.5, 11)	9 (8.75, 10)	9 (9, 9)	0.258
Male gender	5 (29%)	32 (55%)	18 (58%)	0.125
Fever (n, %)	15 (88%)	55 (95%)	31 (100%)	0.179
Fever duration before treatment(d)	3 (2, 6.5)	3 (2, 4)	4 (2, 5)	0.114
Chest radiograph findings (n, %)				0.457
Lobar consolidation	13 (76%)	37 (64%)	23 (74%)	
Patchy	4 (24%)	21 (36%)	8 (26%)	
Pleural effusion (n, %)	6 (35%)	15 (26%)	13 (42%)	0.288
Laboratory findings				
White blood cell(×10^^9^/L)	6.2 (5.15, 7.0)	6.8 (5.5, 7.875)	7.6 (5.6, 8.7)	0.073
Neutrophil (%)	66.5 (56.9, 69.45)	64.35 (58.725, 70.100)	64.8 (53.9, 70.5)	0.947
Platelets (×10^9/L)	229 (179, 262)	212.5 (182.0, 244.5)	203 (181, 256)	0.766
C-reactive protein(mg/L)	17.7 (8.8, 35.9)	13.1 (7.0, 29.75)	17.1 (4.9, 24.5)	0.683
Procalcitonin(ng/mL)	0.081 (0.0515, 0.1375)	0.088 (0.0615, 0.1515)	0.104 (0.710, 0.156)	0.565
Lactate dehydrogenase (U/L)	269 (238.5, 308.5)	274 (244.25, 330.5)	297 (243.5, 334)	0.699

*Comparison between three groups

### Comparisons of clinical courses after therapy

**Fig. 2:**
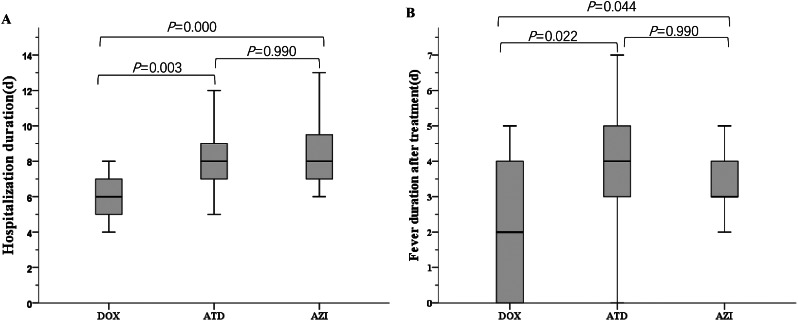
The comparison between the DOX, ATD and AZI groups. (**A**) Hospitalization duration; (**B**) Fever duration after treatment

The efficacy of treatment in each group was compared (Table 2). Hospitalization duration in DOX group was shorter than that in ATD group (6 days vs. 8 days, *P* = 0.003) and AZI group (6 days vs. 8 days, *P* = 0.000) (Fig. 2). The median fever duration after treatment in DOX group was shorter than that in the other two groups (2 days vs. 4 days, *P* = 0.022 and 2 days vs. 3 days, *P* = 0.044, respectively) (Fig. 2). Hospitalization duration and fever duration after treatment were not significantly different between ATD group and AZI group (*P* = 0.867 and *P* = 0.990, respectively) (Fig. 2). The numbers of patients who achieved defervescence within 48 h and chest radiographic improvement after one week of treatment were higher in DOX group than that in in ATD group (*P* = 0.045 and *P* = 0.021, respectively) and AZI group (*P* = 0.000 and *P* = 0.003, respectively). The number of patients using glucocorticoid adjuvant therapy in DOX group was less than that in AZI group (*P* = 0.015). These indicators were no statistically significant differences compared between ATD group and AZI group (*P* > 0.05). Three patients in ATD group received nasal cannula oxygen supply, one patient in AZI group and none in DOX group. No patients were transferred to the intensive care unit or received mechanical ventilation during hospitalization. During intravenous azithromycin treatment, 10 patients had abdominal pain, 3 had vomiting, 3 had rash. All patients treated with doxycycline responded well. No adverse reactions associated with oral doxycycline were observed during treatment.

**Table 2:** Comparisons of clinical courses after therapy in macrolide-resistantMycoplasma pneumoniae pneumonia

	DOX group	ATD group	AZI group	p-value*
	n = 17	n = 58	n = 31	
Hospitalization duration(d)	6 (5, 7)	8 (7, 9)	8 (7, 10)	0.000
Fever duration after treatment(d)	2 (0, 4)	4 (3, 5)	3 (3, 4)	0.020
Defervescence within 48 h (n, %)	8/15 (53%)	10/55 (18%)	1/31 (3%)	0.000
Chest X-ray improvement (n, %)	15 (88%)	30 (52%)	12 (39%)	0.004
Glucocorticoid (n,%)	1 (6%)	16 (28%)	14 (45%)	0.015

*Comparison between three groups

### Comparison of doxycycline treatment in different periods

**Fig. 3:**
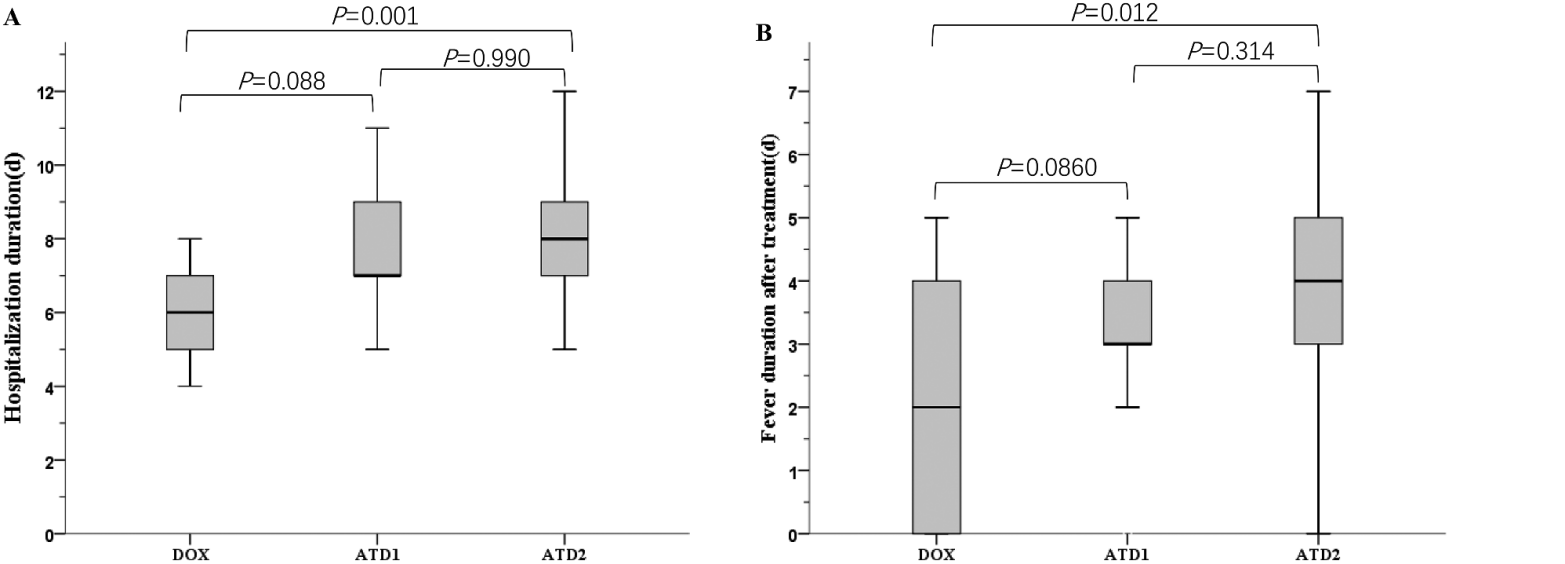
The comparison between the DOX, ATD1 and ATD2 groups. (**A**) Hospitalization duration; (**B**) Fever duration after treatment

As shown in Table 3, the response to azithromycin and doxycycline among children with MR *M. pneumoniae* pneumonia at different time points was analyzed. The median of hospitalization duration and fever duration after treatment were shorter in DOX group than that in ATD2 group (*P* = 0.001 and *P* = 0.012, respectively) (Fig. 3). There was no difference in hospitalization duration and fever duration after treatment in ATD1 group compared with the DOX group (*P* = 0.088 and *P* = 0.860, respectively) and ATD2 group (*P* = 0.990 and *P* = 0.314, respectively). The numbers of patients who achieved defervescence within 72 h were higher in DOX group than that in ATD2 group (*P* = 0.039), and were not significantly different between ATD1 and ATD2 group (*P* = 0.741). The numbers of patients who achieved defervescence within 96 h were higher in DOX and ATD1 group than that in ATD2 group (*P* = 0.024 and *P* = 0.006, respectively), and were not significantly different between DOX and ATD1 group (*P* = 0.990). After one week of treatment, the number of patients who achieved chest radiographic improvement in DOX group and ATD1 group was higher than that in ATD2 group (*P* = 0.003 and *P* = 0.045, respectively). ATD2 group had the highest number of patients using glucocorticoid adjuvant therapy, followed by the other two groups (*P* = 0.007).

**Table 3:** Comparison of efficacy of doxycycline at different time

	DOX group	ATD1 group	ATD2 group	p-value*
	n = 17	n = 17	n = 41	
Age, years	10 (8.5, 11)	9 (8.5, 10.5)	9 (8.5, 10)	0.545
Hospitalization duration(d)	6 (5, 7)	7 (7, 9)	8 (7, 9)	0.002
Fever (n, %)	15 (88.2%)	15 (88.2%)	40 (97.6%)	0.234
Fever duration before treatment(d)	3 (2, 6.5)	3 (1.5, 4)	3 (2, 4)	0.500
Fever duration after treatment(d)	2 (0, 4)	3 (2.5, 4)	4 (3, 5)	0.011
Defervescence within 72 h (n, %)	10 /15(67%)	7/15 (47%)	12 /40(30%)	0.044
Defervescence within 96 h (n, %)	12/15 (80%)	13 /15(87%)	16 /40(40%)	0.001
Chest X-ray improvement (n, %)	15 (88%)	13 (76%)	17 (41%)	0.001
Glucocorticoid(n,%)	1 (6%)	1 (6%)	15 (37%)	0.007

*Comparison between three groups

### The efficacy of early oral doxycycline using PS matched analysis

The efficacy of early oral doxycycline was compared in the matched analysis between the DOX + ATD1group and ATD2 group (Table 4). There was no significant difference in baseline characteristics between the two groups. The hospitalization duration, fever duration after treatment were shorter in DOX + ATD1 group than that in ATD2 group (*P* = 0.037 and *P* = 0.027, respectively). The number of patients achieving defervescence within 72 h was higher in DOX + ATD1 group (*P* = 0.031). More children in ATD2 group were treated with glucocorticoid adjuvant therapy (*P* = 0.002).

**Table 4:** Evaluate the efficacy of early oral doxycycline using PSM analysis

	DOX + ATD1 group	ATD2 group	p-value
	n = 28	n = 28	
Age, years	9 (9, 10)	10 (9, 10)	0.447
Male gender	13 (46%)	16 (57%)	0.422
Fever duration before treatment(d)	3 (2, 5)	3 (2, 4)	0.326
Chest radiograph findings (n, %)			0.783
Lobar consolidation	17 (61%)	18 (64%)	
Patchy	11 (39%)	10 (36%)	
Hospitalization duration(d)	7 (6, 8)	8 (7, 9)	0.037
Fever duration after treatment(d)	3 (2, 4)	4 (3, 5)	0.027
Defervescence within 72 h (n, %)	16 (57%)	8 (29%)	0.031
Glucocorticoid(n,%)	2 (7%)	12 (43%)	0.002

## Discussion

The incidence of MR *M. pneumoniae* has recently increased and has been related to life-threatening or refractory *M. pneumoniae* pneumonia in children [14]. Since the emergence of macrolide resistance has been reported mainly in Asia [7, 15], prevalence of MR *M. pneumoniae* isolated in pediatric patients has increased annually in China [16]: 88.19% in 2016, 90.93% in 2017, 90.56% in 2018 and 92.90% in 2019. In this study, the total number of *M. pneumoniae* pneumonia was 533 cases between May 2019 to August 2022, and 72% (384/533) were MR *M. pneumoniae* pneumonia. The prevalence of MR *M. pneumoniae* was similar to the data reported, and peaked in 2019 during the study period. There was an uneven distribution among cases in the 2019–2022, which was related to the COVID-19 pandemic. Since January 2020, in response to various public health policies to control the spread of COVID-19 in the pandemic, there was a substantial decrease in respiratory infections in China and many resources were focused on diagnosis and management of COVID-19. During the COVID-19 pandemic, our study also showed an increase in MR *M. pneumoniae* infection rates in 2022. The increasing prevalence of MR *M. pneumoniae* has become a significant clinical issue in the pediatric patients. Treatment of MR *M. pneumoniae* pneumonia in children has become challenging.

*M. pneumoniae* lacks cell wall and consequently is resistant to beta-lactams and to all antimicrobials targeting the cell wall [17]. This mycoplasma is intrinsically susceptible to antibiotics that act on the bacterial ribosome and inhibit protein synthesis such as macrolides or tetracyclines or agents that inhibit DNA replication such as fluoroquinolones [18, 19]. MR *M. pneumoniae* is caused by mutations in domain V of the 23s rRNA gene that interfere with the binding of macrolides to rRNA [15]. A-to-G transition mutation at position 2063 in 23S rRNA genes is the most prevalent in MR *M. pneumoniae* isolates, and it is closely followed by the A2064G mutation [2, 3]. Both mutations can cause high-level resistance to erythromycin and azithromycin in *M. pneumoniae* [20]. This suggests that macrolide may have limited effects on MR *M. pneumoniae* infection. Therefore, in cases of MR *M. pneumoniae* strains, alternative antibiotic treatment can be required, including tetracyclines such as doxycycline and minocycline [21, 22]. To date, no tetracycline resistance has been reported in *M. pneumoniae* clinical isolates. Doxycycline has good activity against both macrolide-susceptible and macrolide-resistant strains [22, 23]. As expected, our study found that doxycycline regimens were shown to be more effective than macrolide regimens in patients infected by MR *M. pneumoniae*. The duration of fever and hospitalization were significantly longer in patients with macrolide regimens. Compared to intravenous azithromycin treatment, oral doxycycline is more acceptable to children. Therefore, oral doxycycline is likely to be a better treatment of MR *M. pneumoniae* infections than macrolide for children above the age of 8 years.

The occurrence of MR *M. pneumoniae* infections was likely to lead to treatment failure, which translates into a longer duration of therapy, persistent cough and increased time to resolution of fever compared with treatment-susceptible infection, both in children and in adults [6, 24]. For the treatment of MR *M. pneumoniae* pneumonia presenting clinical and radiological deterioration, adjunctive systemic corticosteroids are sometimes used [25]. However, too early use large doses corticosteroids could cause suppression of phagocytic function of alveolar macrophages and neutrophils, decrease mobilization of inflammatory cells into areas of infection, and cause changes in antigen presentation and lymphocyte mobilization [26]. In addition, corticosteroids did not significantly decrease the DNA load of *M. pneumoniae* in bronchoalveolar lavage fluid [27]. Therefore, untimely corticosteroid additional therapy may increase the risk of mixed infection and they may contribute to condition aggravation [26]. Tetracyclines can inhibit peptide chain lengthening of protein synthesis by acting on the 30 S subunit of *M. pneumoniae* ribosomes. Estimated *M. pneumoniae* amounts after 3 days clearly decreased from 10^6^ copies/mL to 5 × 10^2^ copies/mL in those receiving doxycycline [12]. That indicates doxycycline could decrease the DNA load of *M. pneumoniae*. As indicated by our results in, defervescence occurred within 72 h after initiation of doxycycline (66.7%), with poorer results using azithromycin (ATD1 46.7% and ATD2 30%, respectively). However, when doxycycline was administered within 3 days after azithromycin agents, almost 86.7% of patients showed defervescence within 96 h. Because the inflammatory indicators of our study subjects (such as white blood cells, CRP, LDH, etc.) are usually normal or slightly elevated in the early stages, only a very small number of patients have these inflammatory indicators rechecked after treatment. To avoid selection bias, these indicators were not used as evaluation indicators for efficacy. Due to its ability to inhibit virus replication and reduce viral load, patients receiving oral doxycycline treatment in the early stages can quickly improve clinical symptoms and promote the absorption of pulmonary inflammation. This resulted in a significantly lower number of patients using glucocorticoids as adjunctive therapy in the doxycycline group compared to the azithromycin group. Therefore, clinicians should be vigilant for macrolide treatment failure and consider using alternative drugs if symptoms persist or if there are signs of clinical deteriorations.

Tetracyclines are generally well tolerated, with common adverse reactions observed in patients receiving these agents including anorexia, nausea, vomiting, diarrhea, rash, photosensitivity, tooth discoloration [18, 28]. Most concerning side effect is permanent tooth discoloration. The affinity for mineralizing tissue leads to incorporation into calcifying tissues [29]. However, due to a low affinity for calcium of doxycycline [30], there is no or only negligible tooth staining, even in young children aged 2–8 years [31, 32]. Factors related to tooth discoloration are dosage, duration of treatment, stage of tooth mineralization, and activity of the mineralization process [33]. In our study, oral doxycycline treatment lengths usually range between 7 and 10 days. No adverse reactions associated with doxycycline was observed during treatment. Further studies were needed to evaluate the adverse effects of doxycycline.

This study has some limitations. The first limitation was its retrospective design, which had the potential to introduce memory bias and led to missing data, most notably for assessment of disease severity. Secondly, due to the limitations of the duration of this study, we collected all the qualified children rather than calculated the sample size in the study period. Therefore, it might lead to selective bias. Finally, all research subjects come from one center with limited sample size. Although the PSM method can deal with the issue of selection bias, the small sample size after matching may lead to less objective and complete display of data features. In the future, it is necessary to carry out prospective randomized studies or to conduct studies involving more subjects through multicenter studies.

## Conclusions

Compared with intravenous azithromycin, cases with MR *M. pneumoniae* pneumonia showed significantly shorter duration of fever and hospitalization with oral doxycycline and more rapid improvement of radiologic findings. Most of MR *M. pneumoniae* pneumonia patients achieved rapid defervescence with oral doxycycline or treatment changes to oral doxycycline. Pediatricians should improve the early recognition of MR *M. pneumoniae* pneumonia, which is important for early conversion to doxycycline therapy. Furthermore, a large-scale prospective study is needed to guide appropriate treatment in children with MR *M. pneumoniae*.

## Data Availability

The datasets used and/or analyzed during the current study are available from the corresponding author on reasonable request. All the original data presented in the study are included in the article.
